# Afro-Indigenous Cosmographies of Mobility: Fishes, Viruses and Others Amazonian Lives at the Confluence With the Sars-CoV-19

**DOI:** 10.3389/fsoc.2020.612854

**Published:** 2021-01-15

**Authors:** Stephen Grant Baines, Márcia Leila de Castro Pereira, Potyguara Alencar dos Santos

**Affiliations:** ^1^Department of Anthropology, Universidade de Brasília (UnB), Brasilia, Brazil; ^2^Department of Social Sciences, Universidade Federal do Piauí (UFPI), Teresina, Brazil

**Keywords:** afro-indigenous migratory demography, cosmographies, Brazilian Amazon, SARS-CoV-19, pandemic

## Abstract

The article aims to demonstrate the susceptibility to death that certain Amazonian peoples are facing, as a consequence of their particular migratory demography, which instead of being curbed, have been exponentially intensified by the outbreak of Sars-CoV-19. The article offers an account of the “pendular migrations” and “return migrations” that the indigenous and black rural populations of that region carry out as a result of daily labor displacements, in the search for medical-hospital assistance and the consolidation of political and legal visibility within the cities. In a second effort, directly related to the previous one, we articulate the interference of the viral threat not on the contingency of population flow, but on the dangerous intensification of people circulation between the territorial nuclei of the “first habitation” and the average Amazonian cities, where, as a rule, these peoples maintain “second residences” and to where they regularly transmigrate. To illustrate this phenomenon, three accounts of different Brazilian Amazonian realities are reported: on the rural black population of the banks of the Turiaçu River, Maranhão state, the indigenous people of the savannah *Lavrado*, of the northeast of Roraima state, and the Mura people, who live in the southeast region of Amazonas state. On these realities, the cosmographies of the mobility of their populations are challenged by the changes and strategic conditions imposed by the pandemic.

## Introduction

The article aims to demonstrate the susceptibility to death that certain Amazonian populations are facing, as a consequence of their particular migratory demography, phenomenon that is vertically intensified by the viral outbreak of Covid-19. The article offers an account about the “return migration” that indigenous and black rural populations in the Amazon carry out in the pursuit of their lives, when they seek employment, medical, and hospital assistance and in the consolidation of political and legal visibility. In a second effort directly related to the previous one, we articulate the interference of the viral threat not on the contingency of the population flow, but to the dangerous intensification of transfers between the territorial nuclei of the “first home” and the medium Amazonian cities, where these people maintain “second homes” and for where they regularly migrate.

We bring three cases collected from distinct Amazonian territories. Among the indigenous peoples of the savannah region in the Northeast of Roraima state on the border with Guyana and Venezuela, known locally as the *Lavrado* and *Serras* there are constant flows of people between the villages located on indigenous lands and the city of Boa Vista, the state capital, many of whom have residences on an indigenous land and in the city where they carry out work during part of the year. They commonly spend several months of the year working at their villages, and several months working in Boa Vista, in different jobs, but predominantly as construction workers in the case of men, and domestic servants in the case of women. Those who work in regular jobs in Boa Vista as public servants, in health services, teachers, and a few lawyers and other professionals usually live permanently in the city, but these constitute a small minority.

There are also flows of transnational or cross-border indigenous people between the Republic of Guyana and Brazil, who had their territories divided by the delimitation of the international border in 1904. They have relatives scattered on both sides of the border and transit regularly between villages on both sides, movement limited by the closing of the border since the pandemic started early in 2020. Indigenous people from villages both in Brazil and in Guyana frequently travel to Boa Vista for medical attention in public hospitals.

Another case relates to the period of seasonal abundance of fish in Turiaçu River, located in the central mesoregion of Maranhão state, and illustrates the dramatic confluence between indigenous populations who return to their territories—fleeing from hunger and the virus that ravages the cities—with their “relatives.” From their meeting, versions about morality related to kinship, housing, fishing, and death are updated.

The article also provides an account about the *Mura*, indigenous people who live in villages and also along the rivers and next do the lakes that enter into a complex hydrographic network in the Lower Madeira River, Southern Amazonia. Such villages went through different historical processes in their conformation, based on trajectories of the groups that composed them and that are still doing so. Their strategies of dispersion, circulation and alliance were obviously subject to the ability of each local territorial group to relate to the others. These groups have “well-defined” borders according to the “limits” that they set themselves. They frequently carry out displacements and movements between villages and/or places in a wide circulation area, including the municipalities of Autazes, Careiro Castanho, and Careiro da Várzea. The life of the *Mura* is characterized by mobility. Migration flows, spatial displacements combined with housing mobility and occupational routes and their inflections in time are reflected in the scale of individual and collective destinations.

## The Amazon Territories and the SARS-COV-19

The increase in the number of victims from Covid-19 among the indigenous and black rural populations of the Brazilian Amazon has been acting as a reactive signal to mark the numerous critical challenges involved in achieving the ways of life of native peoples (Milanez and Vida, 2020). If, before the pandemic it was already known that chronic and endemic diseases, the violence of contact relations with the populations of the cities and the pressing threat of hunger caused by the predation of forest resources were menacing the reproduction of traditional Amazonian territories, now, as a result of the pandemic, we stand before a clear threat to the physical, and cultural existence of several of the segments that inhabit the region.

Today, there are peoples who may lose up to 35% of their population due to the special susceptibility of their elderly. Along with the dead, intangible goods like language systems, memorial contents, social and political bonds based on gerontocratic values are under direct threat. Of the 274 languages spoken in Brazil—the remaining sum of the ~1,175 that existed at the beginning of Portuguese colonization in 1500 (Corbera Mori, 2019)—it is estimated that 40 may disappear altogether, because of the knowledge accumulated by the elderly, who are the main victims of the viral threat.

According to the latest epidemiological bulletin systematized by the Special Secretariat of Indigenous Health of the Ministry of Health (SESAI/SUS), which gathers data compiled between May and June 2020, about 23,453 cases of infection, 756 deaths and 188 people directly and indirectly affected by Sar-Cov-19, out of a total of 305 indigenous groups existing in Brazil (Paula and Rosalen, 2020). The bulletin also notes that the evolution of the general curve indicates a weekly average of 1,252 confirmed cases and 25 deaths; which demarcates an intensive growth of contamination among all Brazilian indigenous territories.

In the states of the Amazon region, the Coordination of Indigenous Organizations of the Brazilian Amazon (COIAB) had registered 629 deaths by August 31, 2020. In terms of distribution among groups and villages, the contamination has been presenting a uniform dispersion among groups with different degrees of contact with the cities in the Amazon region. However, due to the characteristics of migratory flows and the absence of areas approved by the Brazilian government that guarantee the territorial accommodation of ethnic groups, as well as the proximity to urban centers with high levels of contamination, groups such as the Xavante, who live in villages in the state of Mato Grosso, have been presenting a lethality rate of 7.09%, more than double that recorded among the non-indigenous population, which is 3.12%, according to the survey of Operation Native Amazon (OPAN). Among the Xavante, the Covid-19 has already victimized 102 indigenous people. The numbers of deaths among the Kokama, Guajajara, Macushi, and Terena ethnic groups are also notably critical.

In the Amazon region there are black rural communities, most of them remnants of enslaved groups that fled from the sugar cane farms. In some territories located in the states of Pará, Amapá, and Maranhão, these “*buissonnières* (forested) communities” (Bona, 2006, p. 3) occupied the areas along the river courses with their settlement nuclei, the *quilombos*; a similar territorial occupation experience to the *Maroons* communities found in Jamaica and Florida and the *Campus* in Guyana and Suriname. For the formation of some of these communities, the rebellious black population joined with the indigenous in the confrontation of the onslaughts of the colonizers against their territories (Gomes, 2005; Klein and Luna, 2010).

**Figure 1:**
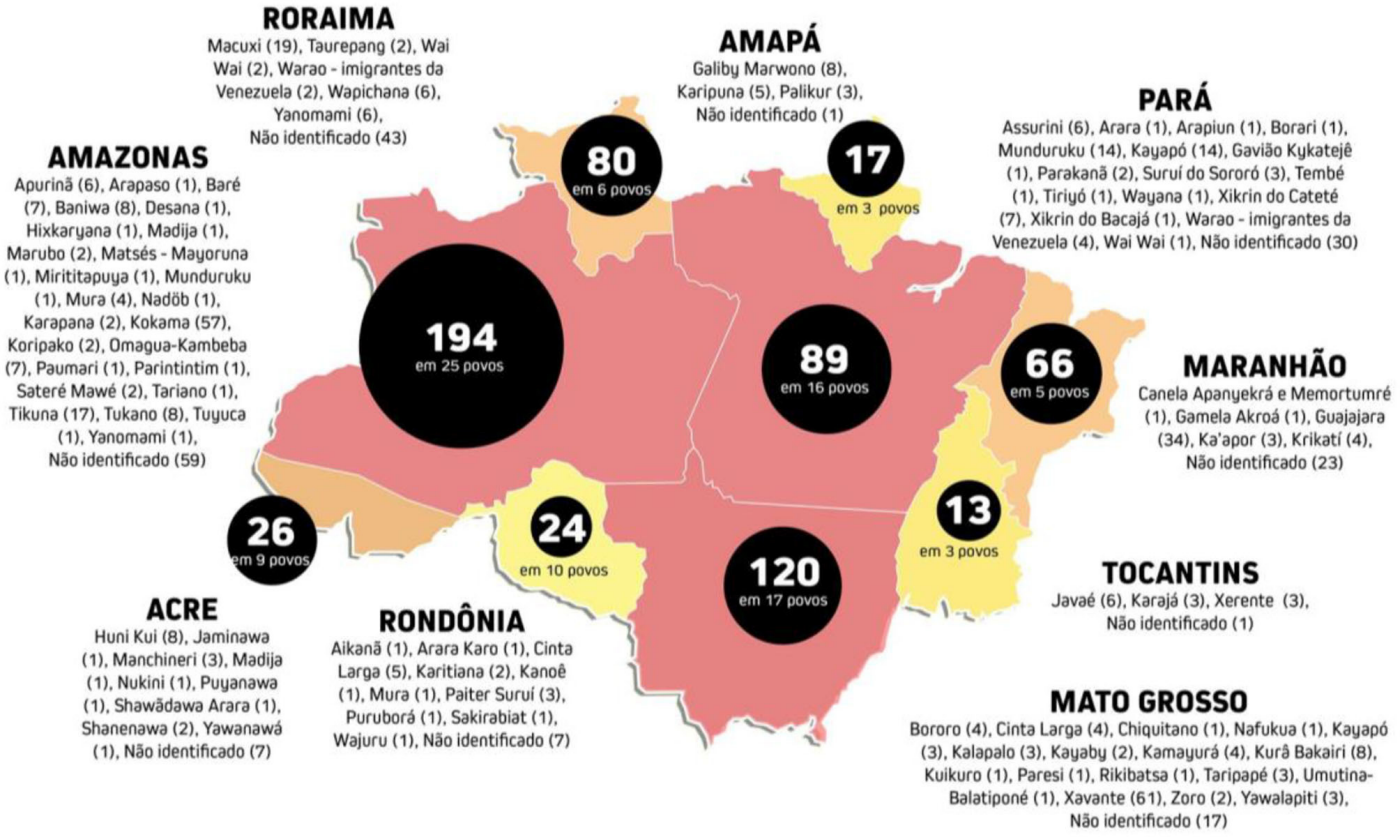
Distribution of the 629 records of death of indigenous by Sars-CoV-19 among the states of the Brazilian Amazon and in 94 groups. Coordination of Indigenous Organizations of the Brazilian Amazon (COIAB). Accessed at: https://coiab.org.br/. Updated on August 31, 2020.

As of September 4, 2020, the National Coordination of Rural Black Quilombola Communities (CONAQ) had registered 156 deaths among the population living in the quilombos, in addition to 3 suspected deaths, 4,541 confirmed cases and 1,214 being monitored. Among the states of the Amazon region, 60 deaths were recorded, with the states of Pará (33 occurrences), Maranhão (09), Amapá (16), Amazonas (01), and Mato Grosso (01) standing out. In light of these numbers, we should mention the impact of underreporting due to the deficit in tests and diagnoses that, in many cases, do not define Sars-Cov-19 as the main causes of death. When we look at the situation of the indigenous and rural black populations as a whole, the scarcity of medical-hospital resources and strategies in preventive medicine exacerbates the problem of underreporting (Figure 1).

## Afro-Indigenous Cosmographies of Amazonian Mobility

When we measure the territorial expansion of the virus within the villages and rural communities of the Amazon, another aspect can be highlighted: the intrusion of the disease among territories which are distant from the cities. There are high notification rates of Covid-19 infections both in villages and communities near cities, and in territories that are days or weeks away from the urban perimeters by boat (Table 1).

**Table 1:** Cases of Sars-CoV-19 infected among the indigenous peoples of the 9 states of the Brazilian Amazon.

**States**	**Suspected cases**	**Confirmed cases**	**Deaths**
Acre	11	2.055	26
Amazonas	103	5.046	194
Amapá	04	1.473	17
Maranhão	0	1.781	66
Mato Grosso	258	2.014	120
Pará	168	4.910	89
Rondônia	153	1.135	24
Roraima	51	2.699	80
Tocantins	47	754	13
**Total**	795	21.867	629

*Coordination of Indigenous Organizations of the Brazilian Amazon (COIAB). Available at: https://coiab.org.br/. Updated on August 31, 2020*.

How could the virus have spread in indigenous and rural black population territories so isolated from the centers of high population concentration of infected people? Answering such a question is one of the aims of this article. First, because for one of the values of Brazilian ethnic and environmental racism—this one based on forging the various “other insiders” of the nation (Pacheco, 2006)–, these peoples have always lived under the sign of spatial isolation and anachronism of “culture” conditions. Secondly, the article seeks to explore further a theme among indigenous and Afro-Brazilian ethnologies within the new context of a pandemic, recognizing that a wide range of work has been published on mobility and migrations, and on the forms of mobility of these groups who are in search of their survival and social reproduction (Teixeira and Brasil, 2008; Sertã et al., 2013; Estanislau, 2014; Pereira, 2016; Arruti, 2019). It is an attempt to set an example for what can be understood as the afro-indigenous Amazonian migratory geography, with a focus on the one produced by the Covid-19 effect. Or, if we want to approach the forms of displacement on which these population movements are based, we could refer to the afro-indigenous cosmographies of Amazonian mobility, as the practical notions of mobility that are implied between

[...] the environmental knowledge, ideologies and identities—collectively created and historically located—that a social group uses to establish and maintain its territory. The cosmography of a group includes its regime of ownership, the affective ties it maintains with its specific territory, the history of its occupation stored in collective memory, the social use it gives to the territory, and the ways it defends it (Little, 2002, p. 4).

**Figure 2:**
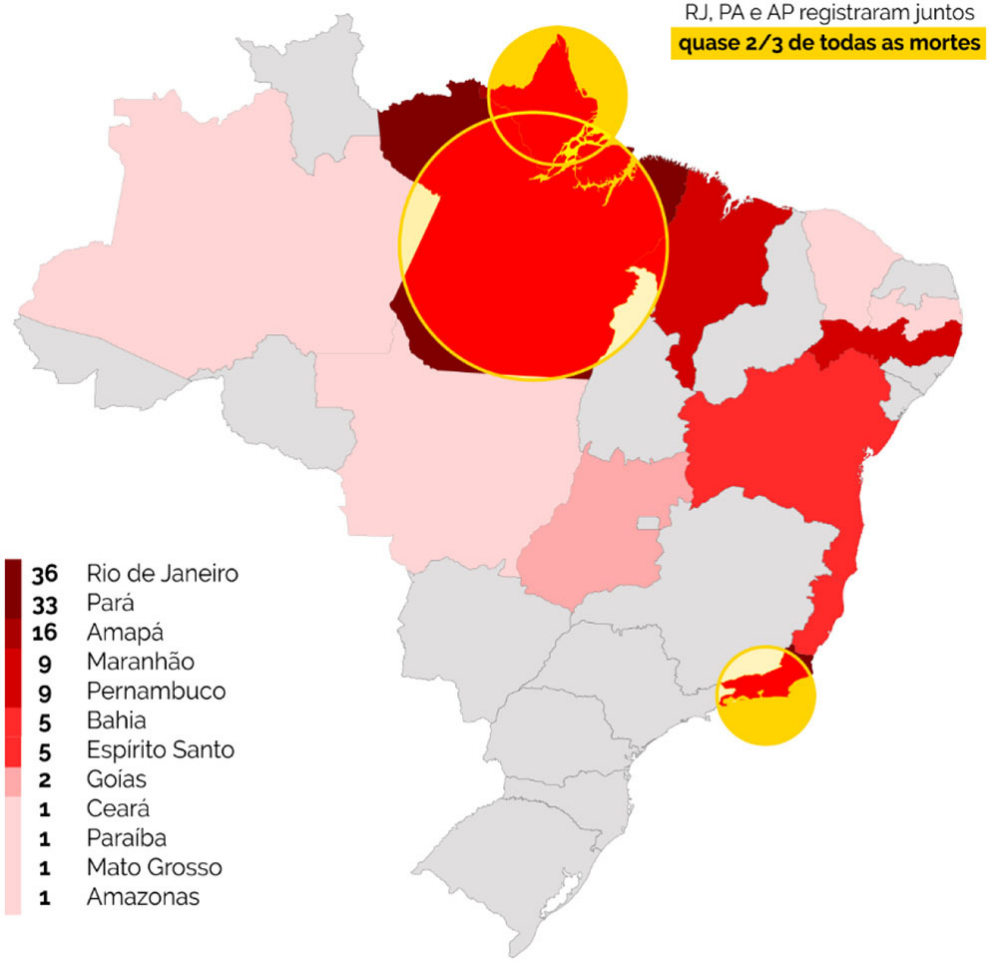
Deaths by Sars-CoV-19 among the *quilombola* population of the Brazilian states, emphasizing the federations of the Brazilian Amazon. Conaq. Accessed at: http://conaq.org.br/. Updated on September 02, 2020.

Unlike the symbolism of immobility or migration based on the false uniformity of the objectives of displacement of those who seek only the production of work links (Durham, 2004), the cosmographies of the mobilities of the afro-indigenous Amazonian populations are based on a series of general and specific demands. Among the territories of the villages and communities and the cities, these populations usually develop countless activities that dispose them in the midst of flexible relationships with urban environments. Thus, among the most common actions are the maintenance of provisional labor relations, the acquisition of semi-processed food, visits to health centers, and the search for visibility and legal assistance to community demands. Unlike the labor migrant known in classical literature, many representatives of the indigenous and black rural populations of the Amazon do not move from their territories to undertake fixed projects of transmigration. Many do not intend to establish a dwelling and produce the displacement of their relatives with them. Instead of maintaining a property as an individual domestic unit in the city, these individuals prefer to share accommodations with relatives or friends who already live in urban areas, because they understand that their stays in these places may be temporary (Figure 2).

The other characteristic of this migratory journey is the relativity of what can be understood as “short and medium distances” between the place of origin and the place of migration. It must be considered, for example, that some villages and communities are at continental distances from the urban centers. Comparatively, it is as if some individuals worked 2 weeks in the south of France and traveled for a week by boat from there to Belgium, where they live. And that this was done several times during the year, until a goal was finally reached, such as working to raise money and buying an engine for one of the village's boats. After that, the migratory journey would be interrupted. Even so, despite these continental distances in the Amazon, these can still be understood as a “pendular migration of short and medium distance” for that population (Teixeira et al., 2009), which ends up making most of their displacements a kind of programmed “short return migration” (Baptista and Campos, 2017).

In carrying out these trajectories of displacement, the Amazonian population with a traditional base economy is compelled by a double impossibility: they cannot abandon the traditional territory at all and live completely apart from the city's resources and services. Even when maintaining their first residence in urban centers, these migrants still make regular use of the agroforestry resources available in their territories: fishes, fruits, cereals, wood, and others. This dependence on the products of forest extractivism regulates the pendular routine of migration between the urban center and the territories, or between the first residence of the original family and the second residence of the temporary stay. Thus, from the point of view of the migratory cosmography of these populations, traditional territory and urban territory are as if “circumstantial places” (Marandola, 2014) fully interconnected and disposed to the conditions of reproduction of life. The jumps between one and the other depend on fortuitous conditions. The endemic and chronic diseases that plague the Amazonian populations—malaria, dengue, hypertension, diabetes and, today, the Covid-19—and that force them to carry out continuous preventive and therapeutic itineraries and to live these experiences “between places” (Ennes, 2010), instances that are accessed depending on certain urgencies and opportunities. In this sense, the place as a circumstance presupposes a

[...] relative eventuality: a position and a situation that emphasizes the relational sense of the being-and-being-in-the-world, while giving due weight to the phenomenal reality of the being-there and its spatiality [...] The path to this reflection will be the idea of the surrounding world (*Umwelt*), as used in the fundamental ontology of Martin Heidegger, and in the ontology of modernity of Anthony Giddens. In the first case, we have the elements to think circumstantiality in its original phatic dimension: the worldliness. In the second case, the surrounding world becomes spheres of protection and senses lived by the self in contemporaneity (Marandola, 2014, p. 230–231).

By this definition, the very biophysical existence with the forest is made of a cosmography of circumstantial places. Ecologies and mobilities make it possible for the Amazonian people to develop fortuitous agreements with the biotic and abiotic entities of the landscapes. In general, life is compartmentalized among the years of strength or decline of resources and conditions: the years of greater elevation of the river waters, when there exists abundance of hunting and fishing, periods of greater or lesser incidence of environmental disasters, conflicts, or epidemics such as malaria (Homma, 1982; Katsuragawa et al., 2008). Thus, the ecological conducts of cohabitation with the forest and with those who exploit it are essentially circumstantial.

In this scenario, Covid-19 would be just another phenomenon competing with the unpredictable challenges that the Amazonian populations have learned to live with over the years. For this reason, it was expected that the Covid-19 would be just another setback to which human collectives would find answers, in order to overcome them with many losses, but with significant accumulated experiences. The surprise brought by the challenge of Covid-19 lies in the lethality of the viral outbreak and the systemic shock caused by the disease. In some cases, representatives of traditional populations that lived in the cities—keeping there their first residences—had to undertake sudden and definitive displacements to the interior of their original territories; sometimes to their families, sometimes to houses that were built in a hurry, in order to welcome them. Fleeing from hunger and disease that compromised life in the cities, these indirect victims of the pandemic ended up increasing the pressures on their territories, because they brought with them demands for forest resources and the very unsuspected existence of the virus carried in their bodies; an epidemic factor that has been causing many deaths in indigenous villages and rural black communities, as shown by the infographics exposed above.

The confluence of these phenomena has demanded the use of a methodological sensibility based on the perspective of “multiactor ethnography” (Bennett, 1993; Little, 2006) employed by some anthropologists and political ecologists to understand the relations between the “Amazonian forest peoples” (Bona, 2006) and the technical, socio-environmental and material challenges that surround their realities. Like some epistemologies that think of the social phenomenon as something composed of “relational multiplicities” of distinct natures—as reflected by “multi-sited ethnography” (Marcus, 1995), the Actor-Network-Theory (Latour, 2007) and methodologies employed by studies on other viral outbreaks in the Amazon (Katsuragawa et al., 2008) –, the focus of multiactor ethnography

[...] is not about the way of life of a social group, but the multiple social and natural interactions that underlie them. Second, it does not deal with a single social group, but with several social groups simultaneously. Third, the geographical scope is less and less limited to the local scope of the group, since it incorporates several levels of social articulations (Little, 2006, p. 92–93).

The researchers developed different strategies and instruments to follow in a procedural and instantaneous way the impact of the pandemic on the territories where they develop their research. In the case of the *quilombola* population of Maranhão, the researcher was undertaking a long period of fieldwork when the first cases contaminated by Covid-19 were registered in the northeast of Brazil. The immediate blockade of interstate highways by the local government prevented him from leaving the territory immediately, which eventually gave him the opportunity to accompany the migratory movements of the quilombola population between the nearby urban centers and the Imbiral Cabeça-Branca territory.

In the case of reports from the Mura populations and the Lavrado region, the researchers resorted to interlocution with indigenous representatives through virtual communication channels and to the large number of reports arriving from those territories by indigenous spokespersons and the websites of the indigenous movements in the Amazon.

The three next reports bring some examples of cosmographies of Amazonian mobility in different regions of Brazilian territory, giving descriptive emphasis to the changes and strategic conditions that have been imposed by the dissemination of Sars-CoV-19.

## Fishes, Viruses and Others Amazonian Lives in Return to the Turiaçu River

Turiaçu is a river that has its course arranged in the western region of the state of Maranhão. Between the XVII and XIX centuries, the river made possible the meeting between Portuguese colonizers, Austro-Hungarian expeditionaries, native groups and the black population fleeing from the agricultural farms of Colonial Brazil (Spix and Martinus, 1828). On the banks of its middle course, the river has a series of settlement nuclei formed by rural black population, the *quilombos*. Some of them, like Imbiral Cabeça-Branca, were formed by old alliances of war between black slaves and Akroá-Gamela native people, who, through armed confrontations against the Portuguese, sought to recover the territories of their ancestors.

In April 2020, when the first cases of contamination began to appear in the city of Pinheiro, which is the closest urban center to Imbiral Cabeça-Branca, the population of that quilombo community was preparing for the beginning of the period of greater abundance of fish in the streams and lagoons formed by the flooding of the River Turiaçu. That was the time of the visit of a series of “relatives” who lived in the city, people who every year used to go to the community in order to take advantage of the abundance of fish in the Turiaçu, an attitude that they call “making a fair in the river”; that is, harvesting from the river only the items of daily subsistence. After the fishing, which was usually done through cooperative work groups, these individuals used to go back to their residences in the cities, leaving behind their relatives.

The viral outbreak of Covid-19 and the occurrence of numerous cases of contamination in the Legal Amazon region of the state of Maranhão created an atypical confluence between events. Instead of making only a temporary return to their communities, many individuals who maintained residence in the city were forced to settle in their original communities, abandoning their houses in urban centers. Thus, they took advantage of the regular period of the abundance of fish, when the migration from the cities to the communities is usually intense, and settled permanently in their home communities. In the cities, many of the job offers had disappeared as a result of the pandemic. Hunger and disease began to rage together within those urban realities.

Provisional employment links in the construction industry, in public works of urban infrastructure and in logging companies that prepare wood for furniture factories are some of the occupational options available to these indigenous and *quilombolas*, who often return to their home territories in precise dates during the annual calendar. These displacements usually accompany the seasonality of the geo-environmental and ecological cycles of the forest: the flood and drought of the rivers, the rainy season that demands the beginning of family vegetable crops and the periods of abundant forest resources are, in general, some of the conditions that mark these programmed returns.

When expelled from the cities by the Covid-19 threat, what these “returnees” found within their original settlement nuclei was a non-receptive environment to their presence. From the point of view of the available resources, the quantity and quality of the goods of the forest were different from those they knew from other times. Over the years, the farms in the region had appropriated portions of land that were essential for the reproduction of the extractive practices of the communities and for the construction of new residences. The main bed of the River Turiaçu saw its fish population decrease year after year, due to the silting up of its course. And the felling of the forest around the communities had produced the resurgence of diseases such as malaria, which had not caused victims in the region for years. When the first cases of Covid-19 infection were reported in the city of Pinheiro, two children had already been diagnosed with malaria in the interior of Imbiral Cabeça-Branca.

From the point of view of moral relations, the return of these individuals to their first residential nuclei established an environment of suspicion as to their presence, due to the imminent risk that they represented, because they could have brought the virus in their bodies. The very territorial pressure for the increase of the population contingent in the communities has triggered an environment of disputes for space and questioning by the authorities of some leaders, especially the older representatives. Almost always, these conflicts revolve around the definition of where a new house should be built: whether within the original family of parents and grandparents or opening a new area within the community's forest reserves. Within most of the rural black communities, the opening of a new house demands, as a rule, the creation of a new productive family plot (Santos, 2010). The demand for a place to live by these “returnees” brought a second challenge to their territories: because the opening of this new productive domestic plot required the cutting down of an area within the native forests that serve community extractive practices. Promoting the opening of clearings like these becomes an attitude of extreme consequences, in a context already marked by the progressive scarcity of forest resources and threats to the balance of social-ecological relations promoted by illegal land trade, deforestation and, more recently, the outbreak of Covid-19.

What the pandemic has been promoting in territorial contexts like these is the moral re-reading of the cosmographies of the mobility of these Amazonian. If before, the journeys between the circumstantial places of the forest and the city were programmed by those who kept residence between territories, now the presence of these returnees is seen with concern by some communities, mainly their leaders. The pandemic has made it evident that, different from the thesis promoted by the Brazilian agro-industrial elite that “there is too much land for too few Indian” (Reydon et al., 2015; Spavorovek et al., 2019), the Amazonian peoples suffer from the scarcity of land and resources, and any demographic fluctuation in the number of inhabitants within their villages and communities can cause ruptures in relationships and real threats to the maintenance of life within these nuclei.

Two months after the information in this report was gathered through a field visit that ended at the beginning of the pandemic, a doctor specialized in indigenous and quilombola collective health who acts voluntarily in Imbiral Cabeça-Branca warned us that the communities of the River Turiaçu were already registering dozens of suspected Sars-CoV-19 infected people. The absence of clinical tests was preventing a precise diagnosis. Some elderly people who were sick, affected by chronic diseases, were trying to keep themselves in isolation, controlling the number of people they kept in contact with—despite the large number of relatives that make up the traditional domestic units –, while the flow of new “returnees” from the cities remained constant.

## The “Time of Sickness” and the Many Wars That the Mura Wage

The indigenous group Mura have surely undergone important changes in their social and political organization, in contact with white populations since the XVIII century. There have been many changes in the settlement patterns of the populations living near the large lakes and along the banks of the channels of large rivers such as Madeira, Amazonas, Purus, and Solimões, by the interiorization of the spaces of their tributaries, smaller rivers, streams and hidden lakes (Menéndez, 1981/1982).

Restricting our geographic view, we will limit it to the Lower River Madeira, more precisely the Autazes Delta, located between the Madeira, Amazon, and Lower Purus rivers. The territory has areas of land and floodplains and, on the occasion of the rising waters, the floodplains are flooded and the various water systems become interconnected, constituting a water network through diffuse and asymmetric channels. The intermediate rivers of the Autazes Delta have interior “paths”: paranás, igarapés, lakes and headwaters, boreholes, entrances of boreholes, and “mouths,” which constitute the essential routes for the movement and synthesize an unlimited network of entrances and exits, whose role is fundamental in the circulation structure of the group. The places used for fishing, the main productive activity of this people, and the availability of fish are directly influenced by the climate and the flood regime in the periodically flooded areas (Junk et al., 1989; Castro and Mcgrath, 2001).

The instability produced by the agropastoral occupation fronts has reached all the lands of these people in the Autazes Delta, although some local groups managed to remain on fractions of land they had previously occupied, others were expelled and, as a result, had to take refuge on lands where the process of land regularization was almost finished. All the dozens of villages today share the situation of harassment, pressure and violence represented by contact with the agropastoralist fronts and large mining projects. They share the same problem: the loss of physical space and the impossibility of exercising displacement to old settlements.

The phenomenon of Mura mobility has been treated by historical literature as errance or nomadism. But we know that the invasions by white populations into the spaces led to an increase in the number of settlements created from internal migrations. The studies or reports point to a discussion often related to movements where the theme of mobility is not problematized, only pointed out as a remnant of an “erratic life” (Ramos, 1997). On the other hand, the territorial configurations of this people are also the result of the process of leaving places, arrivals, and returns.

The stories about the diseases that affected these people are numerous, vivid, and detailed. In the wars they have fought and still fight, they have much to say about the “time of sickness.” According to the memory of the oldest, “in the old days, the Mura ran away, tried to run away from the disease, but it came, it was measles, it was chickenpox, it was smallpox, it was the flu,” as the indigenous Luiz Braga Gomes, Aldeia Murutinga, Rio Mutuca, tells us. Respiratory diseases were once the main cause of death among the Mura, which makes the current pandemic especially dangerous for these people. On April 21, 2020, the first case of Covid-19 was identified in the Murutinga village, in the Murutinga/Tracajá Indigenous Territory, in Autazes, Amazonas. The information caused concern and fear among the indigenous people.

Murutinga is a village, or *aldeados* as they usually call it, densely populated, with a population of around 3,000 people. The territory has always attracted new families, in view of the existence of piped water, from an artesian well, and the arrival of electricity that can be enjoyed for much of the time. Like all Mura villages, Murutinga consists of a variable number of residential units. In its most simplified version, the Mura “places” were made up of domestic groups that were not very fixed, which, as they say, “walked without whereabouts,” that is, they were small groups that made up a floating and mobile population.

Faced with this situation, the leaders suggested adopting social isolation and restricting the exit of indigenous people to fish as a way to prevent the spread of the disease. In this dramatic situation, the pandemic has a direct impact on the food security of these people, since fishing is the main productive activity and the Mura are excellent fishermen. Fish are considered the main source of animal protein and food base throughout the year for families, always seeking the places where the fish is most available according to the hydrological and migration cycles of the fish they know so well.

In any case, the village Murutinga is <2 kilometers from the village of Novo Céu, where there was already information of at least a dozen cases of people infected by Covid-19. In recent decades, the social space occupied by the Mura has undergone a rapid process of demographic, ecological, and economic transformation. Considering that the Mura population has grown expressively in recent decades, thus increasing its proportion of young people, there are serious restrictions in the sphere of physical and cultural reproduction faced by this population. The reduction in areas of use and the strong population growth experienced in recent decades have serious implications for the future of this group.

Nowadays, food production in Murutinga/Tracajá is mainly for subsistence. And, at the same time, the increase in demand for food within the territory means that the relationships between people living on indigenous land and those with others are being modified. Today, the Mura access the commerce of the city of Autazes and Vila do Novo Céu to complement their diet with items that do not produce, and, unfortunately, are places where SARS-coV-19 has been manifested in exponential rhythm. There are other ways of obtaining income, such as daily rates for services (work in third party farms, etc.) provided to non-native people; and public service jobs, hired indigenous teachers and functional positions at FUNASA/SESAI, or informal jobs in the Vila do Novo Céu. According to the website of the Indigenous Missionary Council (CIMI), based on information provided by the indigenous teacher Amélia Braga Cabral, 52, the Murutinga village school was adapted to receive people who move to Autazes, Careiro da Várzea, or Manaus and stay there under quarantine for 15 days: “Today there are pregnant women who had a baby in Autazes. There is also a 55-year-old man in isolation who is suspected of Covid-19. He is being assisted by health workers,” explains Amélia Braga^1^.

We are faced with the perspective that the traditional patterns of Mura social life—dispersion, circulation, articulation and alliance—may be updated in practice, due to the disease, because such patterns will always be subject, obviously, to the capacity of each individual or local territorial group to restrict their movements. In this new “time of sickness,” the infinity of effective possibilities of displacement must be restricted due to the pandemic, however, the moment is also a way to take seriously that wars, illnesses or even recent changes, are tracing other social (dis)ordinances. The trajectories and displacements in which these changes take place may offer indications about the ongoing societal logics and dynamics, such logics will be fundamental in understanding the illnesses among them.

## The Covid-19 Epidemic in the Northeast of Roraima State: a New Chapter of a Long History

As mentioned above, in additional to frequent movements of indigenous people between indigenous lands in Roraima and the capital, Boa Vista, there are also cross-border movements over a vast territory which existed before it was divided by the borders imposed by national States, separating indigenous peoples by national divisions, however, seen from indigenous peoples perspectives as being external impositions of national States. Many have dual nationality, seeing themselves as transnational with the right to free transit across the international borders imposed on their traditional territories (Baines, 2012). Most indigenous people in Roraima, and many who live in the region of Guyana close to the Brazilian border too, depend on their residences in their villages and access to communal Indigenous Lands to produce horticultural products for their own family consumption, as well as selling any excess locally to obtain small amounts of money to buy basic manufactured goods. They also depend on spending periods in their residences in the capital of Roraima, Boa Vista, to earn money through wage labor to gain greater access to industrialized products on which they have now become dependent for several generations. This oscillation between periods spent in their villages and periods spent in the city has been going on over several decades. Also, many families have members who live in Boa Vista and return regularly to their villages to visit their families and spend periods participating in family activities. The closing of the borders early in 2020 has certainly restricted these movements.

Historically, these cross-border movements have followed fluctuations. In the first half of the XX century, when education and health services were seen as being better in Guyana, many indigenous people migrated from Brazil to Guyana, frequently driven off their lands in Brazil by non-indigenous ranchers who invaded their lands, as well as by Brazilian placer miners. After the independence of Guyana in 1966, and the Rupununi Uprising in 1969 when some part Scottish/Indigenous descendant farmers of the Rupununi border region with Brazil threatened to separate from Guyana, an Uprising quickly squashed by the Guyanese army, the central government of Guyana withdrew support for this border region and economic conditions worsened, resulting in a large scale movement from Guyana to Brazil. After Roraima was raised from a Federal Territory to a State in 1988, economic development grew at a very rapid rate as did the urban population of Boa Vista, leading many indigenous people on the Guyana side of the border to see Brazil as an economic giant compared to Guyana, a small country divided by ethnic tensions between its Afrodescendant and East Indian descendant majority populations. In recent years the Guyanese government has started investing in this border region to stimulate economic development.

The border between Brazil and Venezuela presents some specific characteristics, but the indigenous populations which live along this border, have also always transited freely between both countries, having relatives living on both sides of this international border. In recent years this situation has changed very rapidly with the difficult economic conditions in Venezuela and a huge flux of Venezuelan nationals, including many indigenous peoples, to Brazil.

In addition to the cross-border or transnational indigenous people who are largely Macushi and Wapishana, and also some Patamona, Taurepang, Pemon, Yekuana, and others, with the difficulties of life and political tensions in Venezuela in recent years, there has been a massive migration of Venezuelan nationals including indigenous people to Brazil, Warao from the Orinoco delta, about 700 km from the border, in addition to E'ñepa (Panaré), Yanomami and Ye'kuana from the Venezuelan Amazon and Pemon from the savannah region, and people from other indigenous groups from the border region with Brazil, Venezuela and Guyana. Migrants from Venezuela, initially concentrated in the town of Pacaraima on the Venezuelan border, and especially in Boa Vista, capital of Roraima state. The border with Venezuela remains closed since mid-March due to the Covid-19 pandemic, with few irregular entries and exits reported. For individuals already in Brazil, the validity of documentation providing legal stay was extended until the end of the emergency. Indigenous people have faced discrimination as foreigners despite coming under international law as indigenous peoples.

For indigenous peoples, epidemics are nothing new and the Covid-19 pandemic can be seen as a new chapter in a long history of pandemics. Since the Portuguese colonizers occupied the northeast region of what is now Roraima state in the second half of the XVIII century, indigenous people were put into multiethnically composed settlements controlled by colonial soldiers and subjected to a colonial regime, where they were plagued by epidemics of introduced diseases which decimated their populations, The survivors revolted against the colonizers and fled the colonial violence, The anthropologist, Nádia Farage, uses historical sources to reveal that there were a number of uprisings in response to the “overexploitation of the labor of settled Indians” (Farage, 1991, p. 131). Farage also reports massive escapes of indigenous people that spread through these settlements “in proportion to the violence used by the Portuguese to repress them” (Ibid). When repeated attempts to keep indigenous people in settlements on the Rio Branco failed, the Portuguese colonizers began to send them as prisoners to serve as laborers in other regions of the Amazon basin where escape was impossible. Throughout the 19th century, Santilli (2001) and Santilli (1994, 2002) relates the practice of slavery in the region and that throughout the XIX century illegal expeditions continued to capture indigenous people as slaves in this region after indigenous slavery had been abolished in the Amazon basin in 1755. This author affirms that, “slavery continued in the form of private expeditions which relied on the active support of government representatives in the area to recruit indigenous labor force for rubber extraction” (Santilli, 2002, p. 493) in the forests of the lower Rio Branco. The epidemics and revolts eventually led the colonizers to abandon the idea of settlements. Santilli (2002) describes how the slave expeditions in the XIX century were internalized into Macushi cosmology, who saw the non-indigeous colonizers as cannibals who took away indigenous people downriver to the lower River Branco, from where the majority never returned, killed by harsh exploitation and diseases which consumed their bodies.

In the south of Roraima state, the Waimiri-Atroari people also suffered from introduced diseases which swept through their villages decimating their population. Estimates vary as to their exact population in the late XIX century, after two centuries of *Guerras Justas*, the use of armed forces to enslave indigenous people who did not submit to the Portuguese colonial endeavor, and *Descimentos*, the process of invading indigenous villages, capturing indigenous people and transporting them forcibly down rivers to work as slaves in the colonial plantations in Pará and Maranhão, In the second half of the XIX century estimates vary from six thousand to four thousand indigenous people in the Waimiri-Atroari territory (Baines, 1991). By the 1960s their population had been reduced to around 3.000 individuals and with the construction of the BR-174 highway, built under the military dictatorship in the 1970s, which cut straight through the indigenous territory, their population fell to a low point of 332 (Baines, 1991, p. 77) in 1983, of which 216 were under 20 years of age, with a rapid population recuperation in the following decades after they received vaccination campaigns and basic health services.

The decade of 1970 can de characterized by a drastic depopulation, a consequence of conflicts with the Brazilian army which constructed the BR-174 highway, and acted with extreme violence to intimidate the Waimiri-Atroari, but above all by epidemics of introduced diseases with swept through their villages. Some Waimiri-Atroari related massacres perpetrated by a powder which fell from the sky after airplanes and helicopters flew over villages, resulting in many deaths. In the early 1980s, Baines was able to accompany Waimiri-Atroari between 1982 and 1985 over their territory, by then, greatly reduced after dismemberment by a presidential decree to serve the interests of mining companies and the Balbina Hydroelectric Dam which would later flood a vast extension of their traditional lands in 1987. Visiting the clearings of abandoned villages, they related deaths in mass which had occurred over the previous decades, especially during the 1970s when the BR-174 highway was cut through their territory, with indiscriminate contacts between the Waimiri-Atroari and teams of soldiers and contract company workers involved in building the BR-174 highway, bringing epidemics and death. In some village sites, aluminum pots had been perforated, and Waimiri-Atroari explained that they had interpreted the deaths as colonizers' sorcery spread by the industrialized goods received through the interethnic contact. Seeing their relatives die and their society as it used to be, destroyed by mass deaths, they perforated the aluminum pots with anger. In some villages there were no survivors, in other villages a few survived out of a population of an average of 30 to 70 persons in each village. In the early 1980s, transferred to settlements within their own lands administered by the National Indian Foundation (FUNAI), they panicked when there were cold and influenza epidemics, lying in their hammocks fearing imminent death, remembering the traumatic experiences of the previous decade. The last big wave of measles, in April 1991, resulted in the death of 21 Waimiri-Atroari individuals at the Terraplenagem (Yawará) settlement beside the BR-174 highway. When Baines started his field work in 1982, and asked about the other villages, the reply was, “There are no more villages. There are no more of us, we have ended, they all died.” A leader described the large *maryba* initiation rituals, remembering the times with visible happiness and the participation of large numbers of people a few years before, and commented, “Our society (as it was) ended!”

Indigenous peoples in Roraima are among the most vulnerable populations and all are being affected by the Covid-19 with high mortality rates. Up to the 29 September 2020, the Articulation of Indigenous Peoples of Brazil (APIB) estimated that 833 indigenous people had died from Covid and 33.935 had been infected from 158 different indigenous peoples^2^. In Roraima, by late August 2020, the estimate of the Indigenous Council of Roraima (CIR) was of 82 indigenous deaths, especially among the older populations, people who hold the memory, the languages and the traditions of these peoples. The past few months have seen the loss of several important indigenous leaders. To name just a few, known to Baines in his research in the northeast of Roraima where he has been doing field work over the past nineteen years, Alvino Andrade da Silva, a Macushi teacher of 59 years of age died, an indigenous intellectual who had studied philosophy and sociology and participated in the long struggle for the demarcation of the Raposa Serra do Sol Indigenous Land, which was finally confirmed by the decision of the Supreme Federal Court in 2009, after many years of intense work by indigenous people like Alvino. He participated in the creation of the Insikiran Institute for Indigenous Higher Education at the Federal University of Roraima and was coordinator of the “E'ma Pia” Project to guarantee the entry of indigenous students into this university course.

Fausto Silva Mandulão, 58 years old, who lived in the Tabalascada Community, near the capital Boa Vista was a Macushi teacher who also participated in the Insikiran Institute and was working on a new plan for indigenous education in Roraima when he died and had contributed with work on differentiated indigenous education. He was coordinator of the Council of Indigenous Teachers of Amazonia, and member of the State Council of Education of Roraima. He left his wife and five children all with higher education. He completed the Intercultural Bachelor of Arts Program in Social Sciences at the Federal University of Roraima in 2009.

José Adalberto Silva, a Macushi man of 51 years of age, had worked for many years as an activist in indigenous organizations in Roraima and in Brasília, and also participated in the creation of the then Insikiran Nucleus for Indigenous Higher Education in 2001, which later became the Insikiran Institute in 2009, and he was currently Secretary for Indigenous Affairs in the town of Pacaraima on the Venezuelan border.

Dionito José de Souza, a Macushi man of 52, was coordinator of the Indigenous Council of Roraima (CIR) from 2006 to 2011, and participated in the struggle for the demarcation of the Raposa Serra do Sol Indigenous Land and was a key figure in the leadership of the CIR and in the struggle to bring into effect indigenous rights in the state of Roraima.

In Roraima, with a lack of adequate support of the government, many indigenous communities have set up their own health barriers to try to stop the advance of virus into their villages, although the pandemic is affecting many villages despite their efforts. Thirty eight health barriers are mentioned by the CIR on 19/07/2020, which have been installed at access points to communities. Several indigenous organizations have characterized the Bolsonaro government as genocide because of its complete disinterest in indigenous and traditional peoples, and its incentives to invade indigenous lands by the agricultural rural elite and by miners in predatory destructive policies to violently exploit natural resources. The Articulation of Indigenous Peoples of Brazil (APIB) had to approach the Supreme Federal Court (STF) to try to pressure the government to provide basic emergency services as the pandemic advances, Facing a government which is openly hostile to indigenous and other traditional peoples and is refusing to respect their rights, indigenous organizations are adopting a protagonist role in setting up their own sanitary barriers, collecting funds to provide food parcels for those in need, and to buy hospital and protective material to distribute among the indigenous communities.

## A Final Word

In this article, we present three ethnographic realities in which the cosmographies of the mobility of their populations are challenged by the changes and strategic conditions imposed by the pandemic. The spatial arrangements, established historically, have had their possibilities threatened with relevant impediments for various mobility projects. On the other hand, migrations, movements, and displacements, which unite a network of routes and destinations that do not have a single, fixed center, have been characterized, as said, by a dangerous intensification of transfers between the territorial nuclei of the “first home” and medium-sized Amazonian cities, where, as a rule, these peoples maintain “second homes” and where they regularly migrate. Otherwise, the exits, coming and going, for the reasons explained in the article, facilitated the entry of Sars-CoV-19 in the territories. While migration routes have their specific qualities and configurations, depending on the indigenous people, such routes or transfers are located within very wide spatial arrangements. As it is a complex rupture, it has been exceedingly difficult for these peoples to remain totally isolated. Therefore, mobility and restriction operate together for the actors and the forces that operate within the territories.

The three cases from different parts of the Amazon Basin reveal the extremely vulnerable conditions in which indigenous, quilombola and other traditional populations find themselves with the advent of the Covid-19 pandemic. The Afrodescendants in Maranhão state, the Mura people in Autazes, Amazonas, and the indigenous peoples of the Northeast of Roraima state are all subjected to a federal government which has made violent attacks against their constitutional and international rights. Facing deforestation, mining ventures, agribusiness and land invasions encouraged by the government, accompanied by policies to deliberately dismantle both indigenous and environmental legislations, these people are being put in even more vulnerable conditions and left to their own resources and protagonism to face a pandemic which presents a great threat to their futures.

## Data Availability Statement

The raw data supporting the conclusions of this article will be made available by the authors, without undue reservation.

## Author's Note

The article examines the vilnerability of Afro-descendant and Indigenous peoples to the Covid-19 pandemic especially at the time of a government which is against traditional peoples rights and is defending the invasion and occupation of their territories.

## Author Contributions

All authors listed have made a substantial, direct and intellectual contribution to the work, and approved it for publication.

## Conflict of Interest

The authors declare that the research was conducted in the absence of any commercial or financial relationships that could be construed as a potential conflict of interest. The handling editor declared a past collaboration with one of the authors SB.

## Footnotes

^1^Available online at: https://cimi.org.br/2020/05/moradores-da-aldeia-murutinga-am-estao-em-alerta-com-chegada-da-covid-19/ (accessed September 19, 2020).

^2^Available online at: http://emergenciaindigena.apib.info/ (accessed September 29, 2020).
